# Excellent magnetocaloric properties in *RE*_2_Cu_2_Cd (*RE* = Dy and Tm) compounds and its composite materials

**DOI:** 10.1038/srep34192

**Published:** 2016-09-26

**Authors:** Yikun Zhang, Yang Yang, Xiao Xu, Shuhua Geng, Long Hou, Xi Li, Zhongming Ren, Gerhard Wilde

**Affiliations:** 1 State Key Laboratory of Advanced Special Steels & Shanghai Key Laboratory of Advanced Ferrometallurgy & School of Materials Science and Engineering, Shanghai University, 200072, China; 2 Institute of Materials Physics, University of Münster, Wilhelm-Klemm-Straße 10, D-48149 Münster, Germany

## Abstract

The magnetic properties and magnetocaloric effect (MCE) of ternary intermetallic *RE*_2_Cu_2_Cd (*RE* = Dy and Tm) compounds and its composite materials have been investigated in detail. Both compounds undergo a paramagnetic to ferromagnetic transition at its own Curie temperatures of *T*_C_ ~ 48.5 and 15 K for Dy_2_Cu_2_Cd and Tm_2_Cu_2_Cd, respectively, giving rise to the large reversible MCE. An additionally magnetic transition can be observed around 16 K for Dy_2_Cu_2_Cd compound. The maximum values of magnetic entropy change (−Δ*S*_M_^max^) are estimated to be 17.0 and 20.8 J/kg K for Dy_2_Cu_2_Cd and Tm_2_Cu_2_Cd, for a magnetic field change of 0–70 kOe, respectively. A table-like MCE in a wide temperature range of 10–70 K and enhanced refrigerant capacity (*RC*) are achieved in the Dy_2_Cu_2_Cd - Tm_2_Cu_2_Cd composite materials. For a magnetic field change of 0–50 kOe, the maximum improvements of *RC* reach 32% and 153%, in comparison with that of individual compound Dy_2_Cu_2_Cd and Tm_2_Cu_2_Cd. The excellent MCE properties suggest the *RE*_2_Cu_2_Cd (*RE* = Dy and Tm) and its composite materials could be expected to have effective applications for low temperature magnetic refrigeration.

Magnetic refrigeration technology based on the magnetocaloric effect (MCE) shows superior application potential over conventional gas compression/expansion refrigeration technology because of its environmental friendliness, higher energy efficiency as well as compactness^1^,^2^,^3^,^4^,^5^. The MCE is an intrinsic thermal response for the application or removal of a magnetic field to a magnetic material, which can be characterized by the coupled variations of two quantities: the adiabatic temperature change (Δ*T*_ad_) or/and isothermal magnetic entropy change (Δ*S*_M_). To satisfy practical application, extensive efforts have been carried out to pick out the magnetic materials with large/giant MCE as magnetic refrigerants^1^,^2^,^3^,^4^,^5^,^6^,^7^,^8^,^9^,^10^.

Recently, the rare-earth (*RE*) based alloys and oxides, which exhibit the large reversible MCEs and refrigeration capacity with small or zero hysteresis have been of great of interest^11^,^12^,^13^,^14^,^15^,^16^,^17^. Increasing efforts have been devoted for study of the ternary intermetallic compounds of the *RE*_2_*T*_*2*_*X*2:2:1 (*T* = transition metals, and *X* = III group p-metals). Among of the 2:2:1 system, the *RE*_2_Cu_2_*X (X* = Mg, Cd, Sn or In) crystallized with the tetragonal Mo_2_B_2_Fe-type structure^18^, have attracted some attentions because of their unique physical and magnetic properties. The basic crystal chemical data of the different *RE*_2_*T*_*2*_*X* series have been reviewed^19^,^20^. Very recently, Zhang *et al.* and Li *et al.* have reported the large reversible MCEs in *RE*_2_Cu_2_In (*RE* = Dy, Er, and Tm) and Ho_2_*T*_2_In (*T* = Cu and Au) compounds, respectively^21^,^22^,^23^. However, the systems with the p-metals as cadmium are much less known, what besides other reasons could be explained also by the difficulty in materials synthesis due to the high vapour pressure (low boiling point) of cadmium.

To further understand the physical properties of *RE*_2_*T*_*2*_*X* system, in this paper, the magnetic properties and MCE in *RE*_2_Cu_2_Cd (*RE* = Dy and Tm) compounds and its composite materials have been investigated systematically. Not only a large reversible MCE was observed in Dy_2_Cu_2_Cd and Tm_2_Cu_2_Cd compounds, but also an enhanced refrigerant capacity was found in its composite materials.

## Results and Discussion

**Figure 1:**
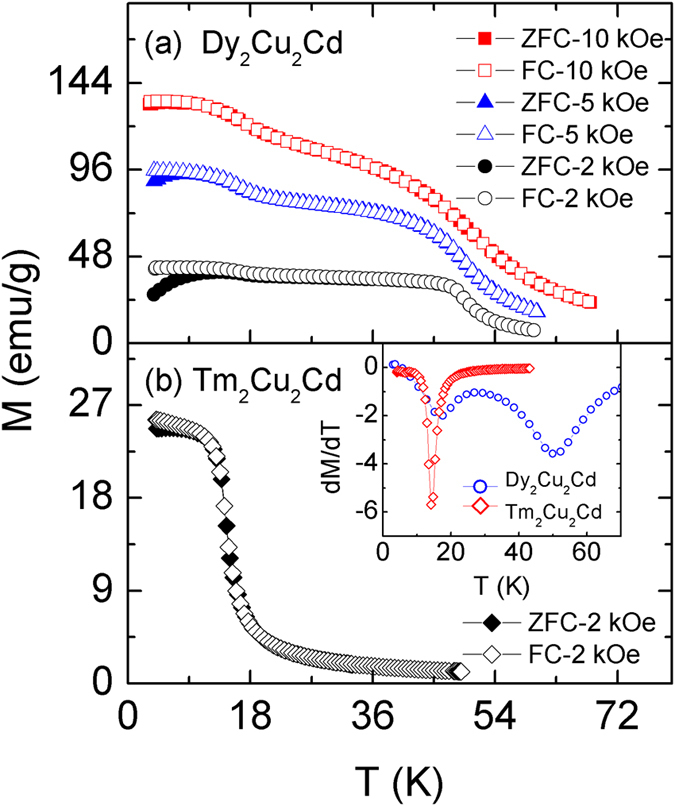
Temperature dependence zero-field cooling (ZFC) and field cooling (FC) magnetization (*M*) under different magnetic fields for Dy_2_Cu_2_Cd (**a**) and the magnetic field of 2 kOe for Tm_2_Cu_2_Cd (**b**) compounds, respectively. Inset of (**b**) shows the temperature dependence d*M*_FC_/d*T* for Dy_2_Cu_2_Cd and Tm_2_Cu_2_Cd compounds under the magnetic field of 2 kOe.

**Figure 2:**
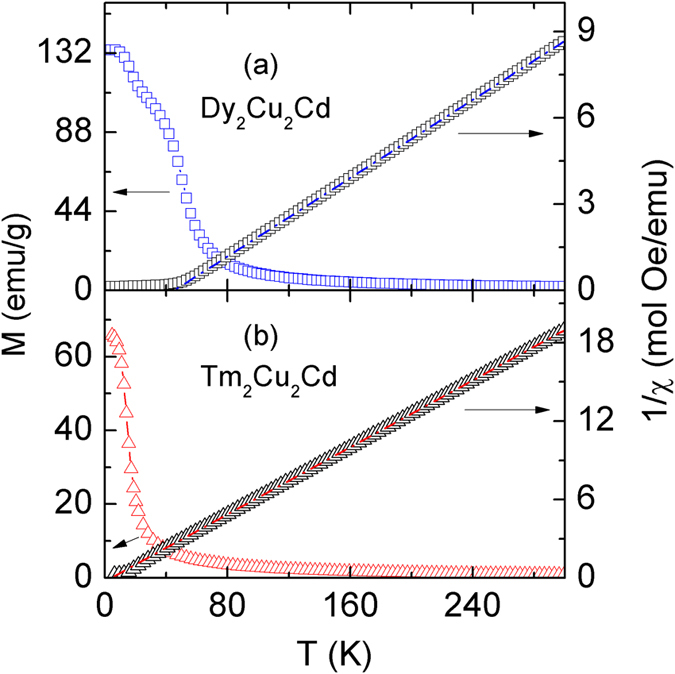
Temperature dependence of magnetization (*M*, left scale) and the reciprocal susceptibility (1/χ, right scale) for Dy_2_Cu_2_Cd (**a**) and Tm_2_Cu_2_Cd (**b**) compounds under the magnetic field of *H* = 10 kOe, respectively.

Figure 1(a,b) show the temperature dependence of the zero field cooled (ZFC) and field cooled (FC) magnetization *M* under different magnetic fields for Dy_2_Cu_2_Cd and a magnetic field of 2 kOe for Tm_2_Cu_2_Cd, respectively. Both compounds display a typical paramagnetic to ferromagnetic (PM-FM) transition, and the Curie temperatures *T*_C_, corresponding to the peak of d*M*_FC_/d*T* - *T* curve [inset of Fig. 1(b)], are determined to be 48.5 K and 15 K for Dy_2_Cu_2_Cd and Tm_2_Cu_2_Cd, respectively. Another magnetic transition can be observed for Dy_2_Cu_2_Cd around *T*_S_ ~ 16 K under low magnetic fields and it shifts to much lower temperatures with increasing magnetic field. Such behaviours may arise from a spin glass transition or spin reorientation phenomenon^24^,^25^, a systematically detail study of the lower temperature magnetic transition will be performed later. The transition temperatures are in good agreement with previously reported values in the literatures^20^. Figure 2(a,b) show the temperature dependence of the magnetization *M* (left side) and the reciprocal susceptibility 1/χ (right side) for Dy_2_Cu_2_Cd and Tm_2_Cu_2_Cd compounds under a magnetic field of 10 kOe, respectively. The 1/χ in paramagnetic regime from 80 to 298 K obeys the Curie-Weiss law for both compounds. The fitted lines are a guide to the eyes for Dy_2_Cu_2_Cd and Tm_2_Cu_2_Cd compounds as shown in the insets of Fig. 2(a,b), respectively. The fit to the Curie-Weiss formula yields positive paramagnetic Curie temperatures (*θ*_P_), *θ*_P_ = 45.3 K for Dy_2_Cu_2_Cd and *θ*_P_ = 14.1 K for Tm_2_Cu_2_Cd, respectively, suggesting dominant ferromagnetic interactions. The effective magnetic moments (*μ*_eff_) are 10.84 *μ*_B_ and 7.72 *μ*_B_ for Dy_2_Cu_2_Cd and Tm_2_Cu_2_Cd, respectively. Such moments are close to those of the free ion values of Dy and Tm taking the theoretical *RE*^3+^ moment of 10.86 *μ*_B_ and 7.56 *μ*_B_, respectively.

**Figure 3:**
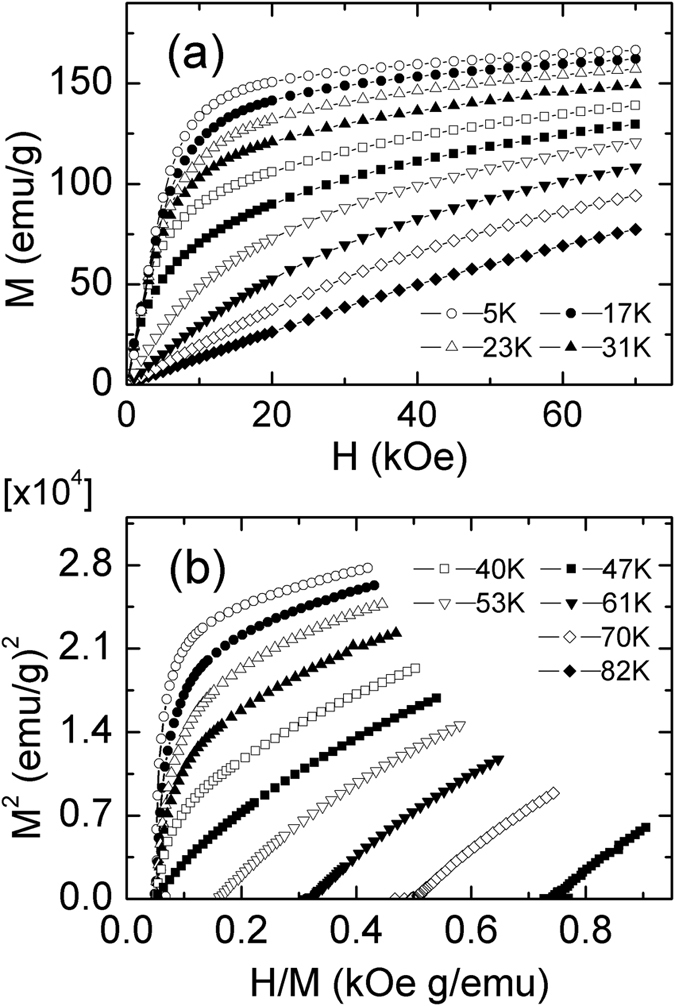
(**a**) Magnetic field dependence of the magnetization (increasing field only) for Dy_2_Cu_2_Cd at some selected temperatures. (**b**) The plots of *H*/*M* versus *M*^2^ for Dy_2_Cu_2_Cd at some selected temperatures.

**Figure 4:**
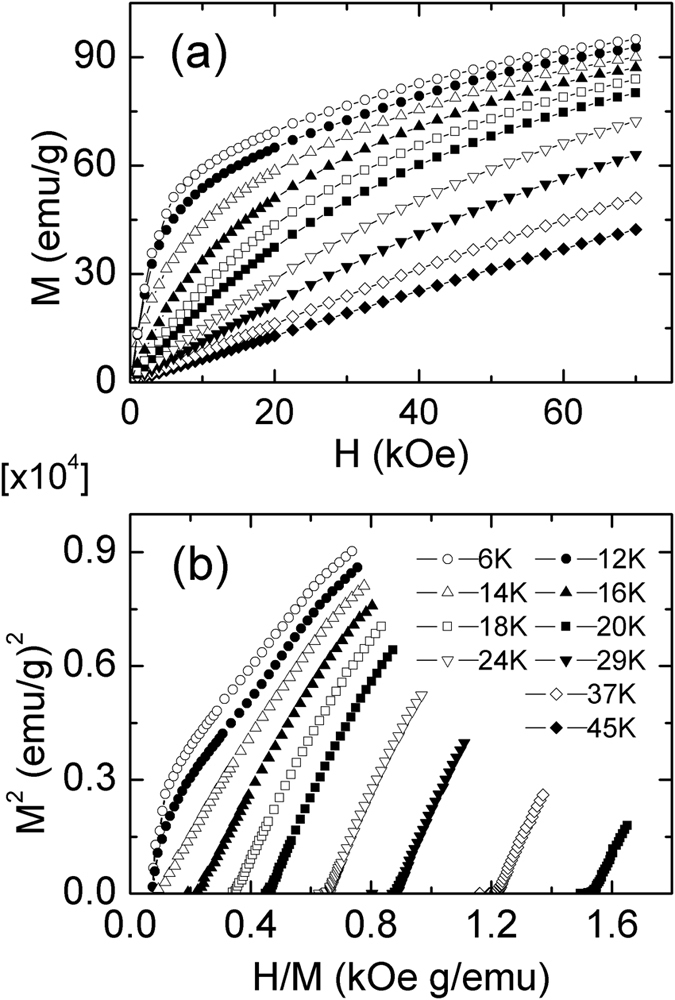
(**a**) Magnetic field dependence of the magnetization (increasing field only) for Tm_2_Cu_2_Cd at some selected temperatures. (**b**) The plots of *H*/*M* versus *M*^2^ for Tm_2_Cu_2_Cd at some selected temperatures.

The magnetic isothermal *M*(*H*) curves of Dy_2_Cu_2_Cd and Tm_2_Cu_2_Cd compounds with increasing field around their transition temperatures with increasing magnetic field up to 70 kOe have been measured and some of them are shown in Figs 3(a) and 4(a), respectively. The magnetization below *T*_C_ increases rapidly in the low magnetic field range for both compounds, and it tends to saturate for Dy_2_Cu_2_Cd compound with increasing magnetic field, whereas it is not saturated at 70 kOe for Tm_2_CuCd compound. To further understand the magnetic transitions, Arrott plots (*H*/*M* vs. *M*^2^) of Dy_2_Cu_2_Cd and Tm_2_Cu_2_Cd compounds are shown in Figs 3(b) and 4(b), respectively. According to Banerjee criterion^26^, the signal (positive and negative) of the slope in Arrott plots has been used to determine the nature of the magnetic phase transition. The negative slopes or inflection points in the Arrott plots often are corresponding to a first order phase transition, whereas the positive slopes are associated to a second order phase transition. By this criterion, neither the inflection points nor negative slopes can be observed in the Arrott plots for Dy_2_Cu_2_Cd and Tm_2_Cu_2_Cd compounds, indicating a characteristic of the second order (FM-PM) magnetic phase transition.

**Figure 5:**
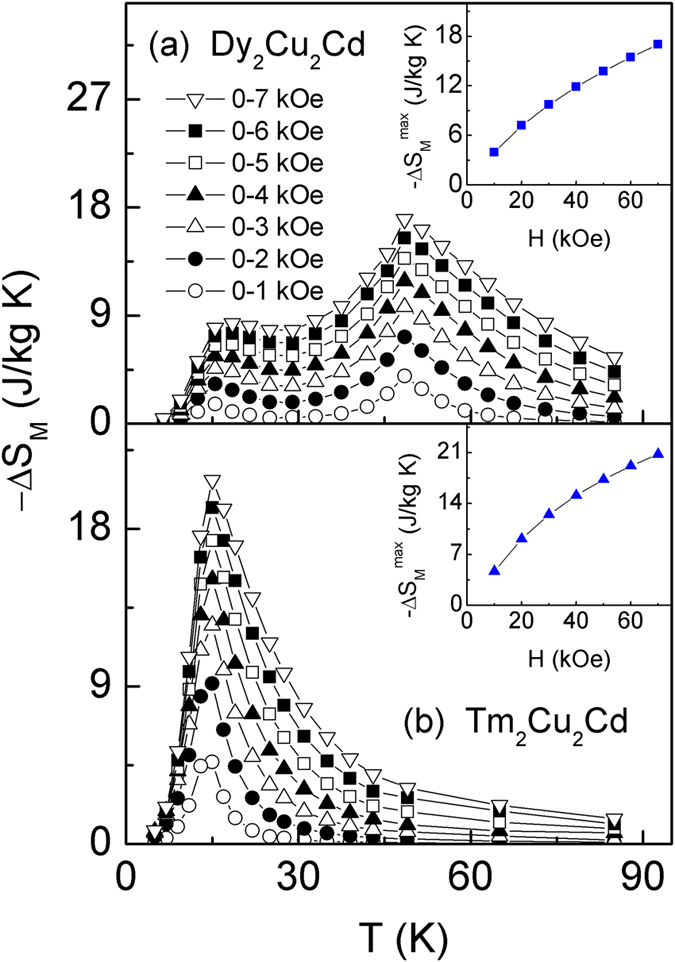
The magnetic entropy change −Δ*S*_M_ as a function of temperature for various magnetic field changes Δ*H* up to 0–70 kOe for Dy_2_Cu_2_Cd (**a**) and Tm_2_Cu_2_Cd (**b**) compounds, respectively. Insets of (**a,b**) show the maximum values of magnetic entropy change (−Δ*S*_M_^max^) as a function of the magnetic field changes for Dy_2_Cu_2_Cd and Tm_2_Cu_2_Cd compounds, respectively.

Figure 5(a,b) show the temperature dependence of magnetic entropy change −Δ*S*_M_ for Dy_2_Cu_2_Cd and Tm_2_Cu_2_Cd compounds which is derived from the temperature and field dependence of the magnetization *M (H*, *T*) by using the Maxwell’s thermodynamic relation^27^, 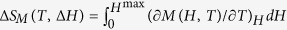, respectively. It can be found that the maximum value of −Δ*S*_M_ increases monotonically with increasing magnetic field change for both compounds [see insets of Fig. 5(a,b)]. Two successive −Δ*S*_M_ peaks (one at around *T*_C_, another at around *T*_S_) can be clearly seen even the low magnetic field change for Dy_2_Cu_2_Cd compound, thus obviously enlarging the temperature range of MCE. Only a pronounced peak in the −Δ*S*_M_(*T*) curves is observed around *T*_C_ for Tm_2_Cu_2_Cd compound. For the magnetic field changes of 0–20, 0–50, and 0–70 kOe, the maximum values of the magnetic entropy change (−Δ*S*_M_^max^) are evaluated to be 7.2, 13.8, and 17.0 J/kg K around *T*_C_, and 3.3, 6.6, and 8.3 J/kg K around *T*_S_ for Dy_2_Cu_2_Cd compound; and to be 9.2, 17.3 and 20.8 J/kg K for Tm_2_Cu_2_Cd compound, respectively.

**Figure 6:**
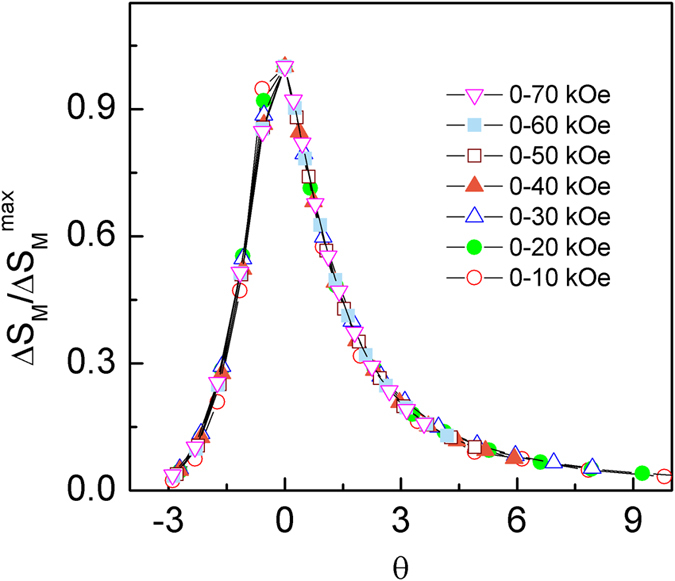
Normalized magnetic entropy change Δ*S′* (=Δ*S*_M_/Δ*S*_M_^max^) as a function of the rescaled temperature *θ* in the present temperature range for Tm_2_Cu_2_Cd compound.

**Figure 7:**
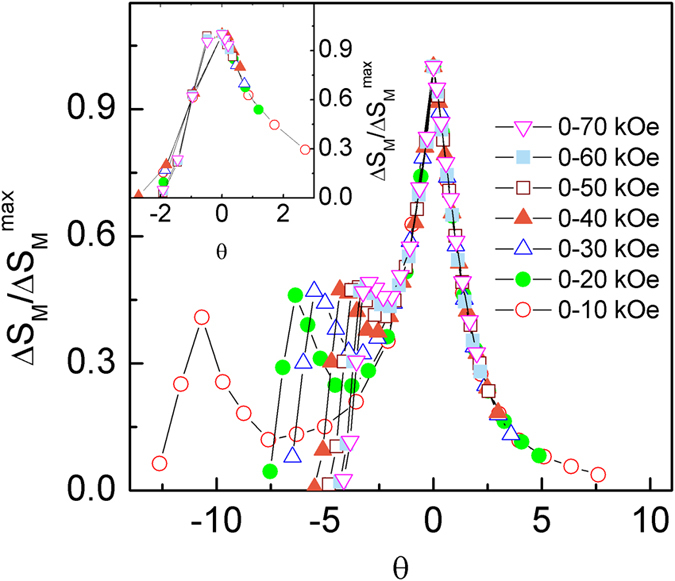
Normalized magnetic entropy change Δ*S′* (=Δ*S*_M_/Δ*S*_M_^max^) as a function of the rescaled temperature *θ* around *T*_C_ for Dy_2_Cu_2_Cd compound. Inset shows the normalized magnetic entropy change Δ*S*′ (=Δ*S*_M_/Δ*S*_M_^max^) as a function of the rescaled temperature *θ* around *T*_S_ for Dy_2_Cu_2_Cd compound.

**Figure 8:**
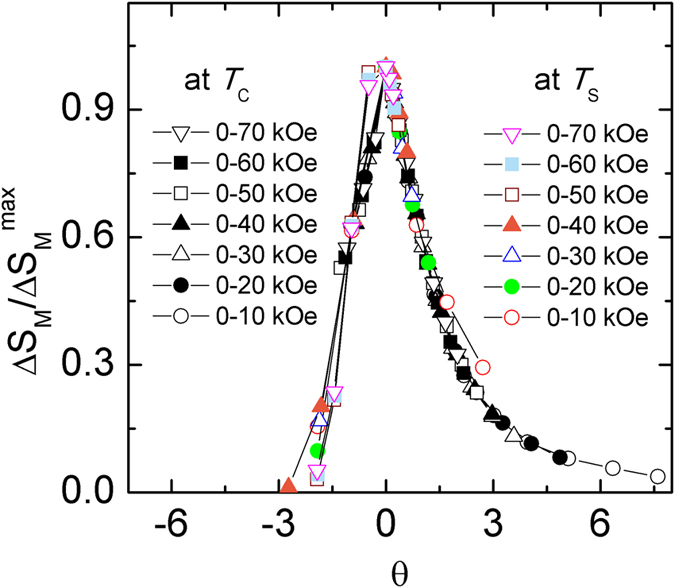
Normalized magnetic entropy change Δ*S*′ (=Δ*S*_M_/Δ*S*_M_^max^) as a function of the rescaled temperature *θ* around *T*_C_ and *T*_S_ for Dy_2_Cu_2_Cd compound.

In addition, the Δ*S*_M_ (*T*) curves for the materials with the second order phase transition can be also described using a universal curve^28^,^29^, which is constructed by normalizing with their respective maximum value Δ*S*_M_^max^ (i. e. Δ*S*′ = Δ*S*_M_ (*T*)/Δ*S*_M_^max^) and rescaling the temperature *θ*, defined as


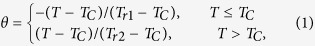


where the *T*_r1_ and *T*_r2_ are the temperatures of the two reference points of each curve that correspond to 0.6Δ*S*_M_^max^. The transformed Δ*S*′ (*θ*) curves for Tm_2_Cu_2_Cd and Dy_2_Cu_2_Cd compounds are displayed in Figs 6 and 7, respectively. We can note that all the rescaled Δ*S*_M_ curves for Tm_2_Cu_2_Cd are overlapped with each other in the present temperature range, as shown in Fig. 6, proving the occurrence of the second order magnetic phase transition in Tm_2_Cu_2_Cd compound. In parallel, the curves for Dy_2_Cu_2_Cd compound are also overlapped with each other around and above *T*_C_ (see Fig. 7). Whereas an obvious deviation below *T*_C_ for *θ* < −2 (around *T*_S_) can be found which is properly due to the spin reorientation phenomenon or spin glass transition. Therefore, the Δ*S*_M_ (*T*) around *T*_S_ (5–30 K) are rescaled and the results are shown in the inset of Fig. 7. Similarly, the curves around *T*_S_ are well overlapped with each other. Furthermore, the rescaled Δ*S*′ (*θ*) curves around *T*_C_ and *T*_S_ for Dy_2_Cu_2_Cd compound under various magnetic field changes are summarized together (as given in the Fig. 8). One can find that all the rescaled Δ*S*_M_ curves can collapse onto one universal curve, which is consistent with the previous investigations that the materials with successive magnetic phase transitions^22^,^24^,^30^,^31^. The analysis of the universal behaviour further confirms that the Dy_2_Cu_2_Cd compound with the second order phase transition.

**Figure 9:**
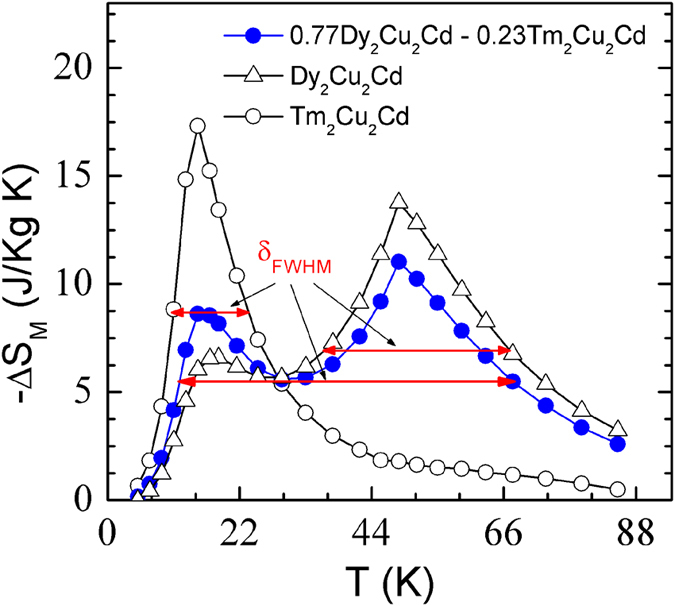
Temperature dependence of magnetic entropy change −Δ*S*_comp_ for the 0.77 Dy_2_Cu_2_Cd - 0.23 Tm_2_Cu_2_Cd composite material for the magnetic field change of 0–50 kOe.

Another important quality factor of refrigerant materials is the refrigerant capacity [*RC*, defined as numerically integrating the area under the −Δ*S*_M_ - *T* curve at full width of half maximum (δ_FWHM_) of the −Δ*S*_M_ peak as the integrating limits]. For the magnetic field changes of 0–20, 0–50, and 0–70 kOe, the values of *RC* are evaluated to be 87, 316, and 495 J/kg for Dy_2_Cu_2_Cd compound; and to be 60, 165, and 248 J/kg for Tm_2_Cu_2_Cd compound, respectively. It is well known that magnetic refrigeration systems based on an ideal Ericsson cycle requires a magnetocaloric material with a constant Δ*S*_M_ over an operating refrigeration temperature range^32^,^33^. Besides the materials with successive magnetic transitions or with a very magnetic field sensitive magnetic phase transitions^22^,^24^,^30^,^31^,^34^, composite materials have been considered to be the most promising method to accomplish the requirement of Ericsson cycle since it can lead to almost constant Δ*S*_M_ with enlarged temperature span^35^,^36^,^37^,^38^. An enhanced *RC* have been successfully realized in Eu_8_Ga_16_Ge_30_-EuO^36^, amorphous FeZrB(Cu)^37^, and ErNiBC-GdNiBC^38^ composite materials. We can note that the Dy_2_Cu_2_Cd and Tm_2_Cu_2_Cd compounds possess the same crystal structure with similar lattice parameters and similar magnitudes of the magnetic entropy change (−Δ*S*_M_). Therefore, these composite materials could be expected to fulfil the required Ericsson cycle conditions. The total magnetic entropy change of *x* Dy_2_Cu_2_Cd + (1 − *x*) Tm_2_Cu_2_Cd composite materials, Δ*S*_comp_(*T*, *H*, *x*), can be calculated theoretically from the individual Δ*S*_M_(*T*) curves^35^,^36^,^37^,^38^,


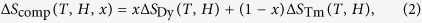


where *x* and 1 − *x* are the weight amounts of Dy_2_Cu_2_Cd and Tm_2_Cu_2_Cd, respectively. Based on both compounds, a composite material can be formed and the optimum ratio of *x* ~ 0.77 is determined by using a numerical method. The magnetic entropy change Δ*S*_comp_(*T*) for Dy_2_Cu_2_Cd - Tm_2_Cu_2_Cd composite material at *x* ~ 0.77 under a magnetic field change of 0–50 kOe is shown in Fig. 9. A table-like MCE in a wide temperature span of 10–70 K can be observed in Δ*S*_comp_(*T*) curve which is desirable for an ideal Ericsson-cycle magnetic refrigeration. The corresponding maximum value of *RC*_comp_ is 417 J/kg, which is 32% and 153% higher than those of Dy_2_Cu_2_Cd (316 J/kg) or Tm_2_Cu_2_Cd (165 J/kg). The transition temperature *T*_C_, the maximum values of −Δ*S*_M_^max^ and *RC* under the magnetic field change of 0–50 kOe for Dy_2_Cu_2_Cd and Tm_2_Cu_2_Cd as well as the 0.77 Dy_2_Cu_2_Cd - 0.23 Tm_2_Cu_2_Cd composite material together with some MCE materials in the similar working temperature range are listed in Table 1 for comparison. The MCE parameters for the present studied materials are comparable or larger than those of other potential magnetic refrigerant materials in the similar temperature region, suggesting *RE*_2_Cu_2_Cd composite materials could be a promising candidate for magnetic refrigeration for Ericsson cycle in the temperature range of 10–70 K. The present results allow for the possibility of using *RE*_2_Cu_2_Cd compounds to fabricate composite materials with desirable magnetocaloric properties for active magnetic refrigeration.

## Conclusions

In summary, two single phased Dy_2_Cu_2_Cd and Tm_2_Cu_2_Cd compounds have been fabricated and the magnetism and magnetocaloric effect have been investigated experimentally. Both compounds undergo a paramagnetic to ferromagnetic transition at their own Curie temperatures, additionally, another magnetic transition is also observed for Dy_2_Cu_2_Cd at low temperatures. For a magnetic field change of 0–50 kOe, the maximum values of magnetic entropy change (−Δ*S*_M_^max^) are 13.8 J/kg K around *T*_C_, and 6.6 J/kg K around *T*_S_ for Dy_2_Cu_2_Cd; and 17.3 J/kg K for Tm_2_Cu_2_Cd, respectively. The rescaled entropy change Δ*S*_M_ curves around *T*_C_ follow a universal behaviour for Dy_2_Cu_2_Cd and Tm_2_Cu_2_Cd, which further confirm both compounds with the second order phase transition. A table-like MCE from 10 to 70 K and a strong enhancement of *RC* have been found in theoretically calculated Dy_2_Cu_2_Cd-Tm_2_Cu_2_Cd composite materials. The maximum value of *RC*_comp_ is 417 J/kg in the 0.77Er_2_Cu_2_Cd - 0.23Tm_2_Cu_2_Cd composite material for a magnetic field change of 0–50 kOe, which is obviously larger than those for either Dy_2_Cu_2_Cd (316 J/kg) or Tm_2_Cu_2_Cd (165 J/kg) compounds. The results indicate that the *RE*_2_Cu_2_Cd (*RE* = Dy and Tm) compounds and its composite materials could be promising candidates for magnetic refrigeration in the temperature range of 10–70 K. Furthermore, the present results may also provide a cost-effective strategy for exploring suitable refrigeration candidates with table-like magnetocaloric feature by a materials composition method, beneficial for Ericsson-cycle in the wide temperature range.

## Methods

The Dy_2_Cu_2_Cd and Tm_2_Cu_2_Cd polycrystallize samples were fabricated by induction melting the elements in a sealed quartz crucible. Firstly, high purity Dy, Tm, Cu and Cd with stoichiometric amounts were weighted and placed in the quartz crucible. Secondly, a high vacuum better than 2*10^−5^ mbar was achieved in the crucible. Then the crucible was filled with purified argon gas at pressure of ca. 750 mbar and sealed immediately. Finally, the quartz crucible was placed in an induction furnace and heated at 1100 K for 4 minutes, following by 3 hours annealing at 850 K. The powder X-ray diffraction (Bruker D8 Advance) measurements were carried out at room temperature using Cu *K*α radiation. Both samples were proved to be single phase, and the lattice parameters were evaluated to be *a* = 7.491 and *c* = 3.742 Å for Dy_2_Cu_2_Cd; and to be *a* = 7.439 and *c* = 3.687 Å for Tm_2_Cu_2_Cd, respectively. The magnetic measurements were performed by using a commercial vibrating sample magnetometer (VSM) which is an option of the physical properties measurement system (PPMS-9, Quantum Design) in the temperature range of 3–298 K with a DC magnetic field from 0 to 7 T, and the samples are small particles of 4.5 and 3.8 mg for Dy_2_Cu_2_Cd and Tm_2_Cu_2_Cd, respectively.

## Additional Information

**How to cite this article**: Zhang, Y. *et al.* Excellent magnetocaloric properties in *RE*_2_Cu_2_Cd (*RE* = Dy and Tm) compounds and its composite materials. *Sci. Rep.* **6**, 34192; doi: 10.1038/srep34192 (2016).

## Figures and Tables

**Table 1:** The transition temperature *T*_C_, the maximum values of magnetic entropy change −Δ*S*_M_^max^ and refrigeration capacity *RC* under the magnetic field change of 0–50 kOe for Dy_2_Cu_2_Cd and Tm_2_Cu_2_Cd as well as the 0.77Dy_2_Cu_2_Cd - 0.23Tm_2_Cu_2_Cd composite material together with some MCE materials with the *T*_C_ from 10 to 70 K.

Material	*T*_C_ (K)	−Δ*S*_M_^max^(J /kg K)	*RC*(J/kg)	Ref.
Dy_2_Cu_2_Cd	48.5/16	13.8	316	present
Tm_2_Cu_2_Cd	15	17.3	165	present
0.77Dy_2_Cu_2_Cd-0.23Tm_2_Cu_2_Cd		11.0	417	present
DyNi_2_B_2_C	10	17.1	~182	39
ErAgAl	14	10.5	~196	40
Dy_2_CoGa_3_	17	10.8	252	41
Ho_2_Au_2_In	21	12.9	~261	23
HoPdIn	23	14.6	~372	24
TbCo_3_B_2_	28	8.7	~215	42
Ho_2_Cu_2_In	30	17.4	~320	23
EuAuGe	33	7.6	~269	43
Tm_2_Cu_2_In	39.4	14.4	260	21
EuAuZn	52	9.1	~239	44
Tb_3_Ni_6_Al_2_	57.5	9.8	~346	45
Dy_12_Co_7_	64	10.0	299	46
